# Nitric Oxide Affects ERK Signaling through Down-Regulation of MAP Kinase Phosphatase Levels during Larval Development of the Ascidian *Ciona intestinalis*

**DOI:** 10.1371/journal.pone.0102907

**Published:** 2014-07-24

**Authors:** Immacolata Castellano, Elena Ercolesi, Anna Palumbo

**Affiliations:** Laboratory of Cellular and Developmental Biology, Stazione Zoologica Anton Dohrn, Villa Comunale, Naples, Italy; Laboratoire Arago, France

## Abstract

In the ascidian *Ciona intestinalis* larval development and metamorphosis require a complex interplay of events, including nitric oxide (NO) production, MAP kinases (ERK, JNK) and caspase-3 activation. We have previously shown that NO levels affect the rate of metamorphosis, regulate caspase activity and promote an oxidative stress pathway, resulting in protein nitration. Here, we report that NO down-regulates MAP kinase phosphatases (mkps) expression affecting positively ERK signaling. By pharmacological approach, we observed that the reduction of endogenous NO levels caused a decrease of ERK phosphorylation, whereas increasing levels of NO induced ERK activation. We have also identified the ERK gene network affected by NO, including *mpk1*, *mpk3* and some key developmental genes by quantitative gene expression analysis. We demonstrate that NO induces an ERK-independent down-regulation of *mkp1* and *mkp3*, responsible for maintaining the ERK phosphorylation levels necessary for transcription of key metamorphic genes, such as the hormone receptor *rev-erb* and the van willebrand protein *vwa1c*. These results add new insights into the role played by NO during larval development and metamorphosis in *Ciona*, highlighting the cross-talk between different signaling pathways.

## Introduction

Metamorphosis, the spectacular post-embryonic transformation of a larva into a juvenile, is a widespread process occurring in many metazoa from amphibians, fishes to urochordates, insects and other invertebrates, allowing the transition of the animal from vegetative to reproductive life stage. In marine organisms, this transition requires that the swimming larvae acquire the competence to receive specific environmental cues, inducing the settlement and the initiation of metamorphosis. The binding of environmental cues to cell surface receptors on the larval sensory organs transmits signals via the nervous system to activate molecular pathways that drive the subsequent morphogenetic events, implying an extensive transformation of the body plan with disappearance of larval structures and appearance and or remodeling of some organs in the adult [1]. An important signaling molecule involved in this process is nitric oxide (NO), which affects key decisions of life and death, by shutting on or off apoptotic pathways and regulating the timing of life cycle transitions [2].

The ascidian *Ciona intestinalis* provides an excellent experimental system to investigate molecular signaling pathways involved in metamorphosis [3]–[5]. In particular, the simplicity of the ascidian tadpole, the rapid rate of development, the availability of genome sequence [6] and the extensive *in situ* gene expression profiles during embryogenesis [7] make the ascidian a suitable organism to characterize the gene regulatory network that controls the onset of metamorphosis. Many studies have described in detail the morphological changes occurring during *Ciona* metamorphosis. After hatching, approximately 18 hours post fertilization (hpf), larvae swim for few hours, during which they acquire competence to respond to environmental cues. Then, larvae stick to a suitable substrate by adhesive papillae and metamorphosis begins (approximately 28 hpf) through a profound reconstruction of the body plan and a remarkable regression of the tail [3], [4]. Adhesive papillae represent specialized organs for metamorphosis as this process is hampered in papillae-cut larvae and in mutants in which the functionality of papillae is compromised [8]. At the molecular level, several processes are involved in *Ciona* metamorphosis: 1- the production of NO [9]; 2- the activation of two members of MAP kinase proteins, the extracellular-signal-regulated kinase, ERK, and the c-Jun NH(2)-terminal kinase, JNK [10], [11]; 3- the activation of the apoptosis-related peptidase caspase-3 [10]. All these events interplay together leading to metamorphosis through a massive apoptosis, starting at the extremity of the tail and propagating all along the body to all tissues [5], [9], [10], [12].

In *C. intestinalis*, NO is produced by a NO synthase (NOS), showing the structural features of a neuronal NOS [13]. The spatial patterns of *NOS* expression, as well as NO detection, are very dynamic: in a few hours both signals move rapidly along the larval body, from the anterior part of the trunk, laterally to future palps, to the central nervous system and tail epidermis, to be finally detected in juvenile digestive organs [9]. In the tail, NO appears also in muscle and notochord cells due to gas diffusion. At the beginning of tail regression, NO is present at the tail extremity of larvae where the apoptotic wave originates. Recently, we have demonstrated that NO also promotes a signaling pathway associated with oxidative stress during *Ciona* development [14].

ERK and JNK play both pro-survival and pro-apoptotic roles depending on the cell type and cellular environment. In *Ciona*, activation of ERK and JNK is necessary for apoptosis and metamorphosis as both processes are completely blocked when these kinases are inhibited [10], [11]. Moreover, the gene network controlled by these kinases has been identified [11]. In particular, ERK is activated just a few hours after hatching (first peak at 20–22 hpf) in the papillae of swimming larvae and later at metamorphosis in tail cells (second peak at 28 hpf) before the wave of apoptosis occurs, suggesting that the phosphorylated form of ERK transduces the apoptotic signal to tail tissues during metamorphosis [10], [11].

Moreover, ERK and P-ERK are found to be nitrated during larval development, suggesting that NO and reactive nitrogen species levels can affect metamorphosis. Indeed, a decrease of NO or reactive nitrogen species levels by NOS inhibition or by NO scavengers markedly reduces the rate of metamorphosis, whereas NO donors or peroxynitrite cause an opposite effect [14].

The caspase-3 protein is a member of the cysteine-aspartic acid protease (caspase) family, whose sequential activation plays a key role in the execution-phase of cell apoptosis. In *Ciona*, the apoptotic wave, responsible for tail regression is dependent on caspase-3 activation and this event is controlled by NO levels [9], [10].

In proceeding with our studies of NO signaling during *C. intestinalis* metamorphosis, we have focused our attention on ERK, recently recognized as a NO target [14] and whose localization during metamorphosis, first in papillae and later in the tail, overlaps the NO signal from the anterior part of the larva to the tail [9], suggesting a cross talk between the two signals. By pharmacological approaches, we now report that modulation of endogenous NO levels in the ascidian larva affects ERK phosphorylation. We have also identified the gene network affected by NO, including ERK dual specific MAP kinase phosphatases (*mkps*), which provide a negative regulation on ERK activity, and some developmental genes whose expression is influenced by ERK phosphorylation.

## Materials and Methods

### Animal handling and incubation experiments

Adult *C. intestinalis* were collected at Fusaro Lake in the district of Naples (40° 49′ 10.6″ north latitude, 14° 0.3′ 32″ east longitude). No specific permissions were required for this location that it is not privately-owned nor protected in any way. The study did not involve endangered or protected species, and was carried out in strict accordance with European (Directive 2010/63) and Italian (Decreto Legislativo n. 116/1992) legislation for the care and use of animals for scientific purposes. Animals were transported to the service Marine Resources for Research and maintained at 18°C in tanks with circulating sea water and under constant light to allow gametes accumulation. Animal handling and fertilization were carried out as previously described [9], [14]. In brief, eggs from a single animal were fertilized with a mixture of sperms obtained from different individuals. Embryos were cultured at 18°C in 0.2 µm filtered sea water. Just hatched larvae were obtained at about 18–20 hpf at 18°C. Development was followed on live specimen with an Olympus stereomicroscope. Samples at appropriate stages were identified using the morphological criteria previously reported by Chiba [15] and were selected on the basis of at least 95% homogeneity. Hatched larvae (about 100 larvae/ml) were treated in tissue culture dishes in 50 ml of sea water at 18°C with the following drugs, at the final concentrations indicated in the text. These include the NOS inhibitor (1-(2-trifluoromethylphenyl) imidazole) (TRIM) (Sigma), the slow releasing NO donor (Z)-1-{N-(3-Aminopropyl)-N-(4-(3-aminopropylammonio) butyl)-amino}-diazen-1-ium-1,2-diolate) (spermine NONOate, sperNO) (Alexis), spermine (Sigma), the NO scavenger (2-(4-carboxyphenyl)-4,4,5,5-tetramethylimidazoline-1-oxyl-3-oxide) (c-PTIO) (Alexis), which reacts stechiometrically with NO [16], the MEK inhibitor U0126 (Calbiochem) and the dual specificity protein phosphatase 1/6 Inhibitor (dusp 1/6 I) (Calbiochem). Stock solutions of 1 M TRIM (in DMSO), 0.1 M sperNO (in 0.01 M NaOH), 0.1 M spermine (in sea water), 0.1 M c-PTIO (in sea water), 0.025 M U0126 (in DMSO) and 0.032 M dusp 1/6 I (in DMSO) were prepared and diluted to the final experimental concentration. The experiments were performed at least in triplicate (see figure legends for details). In the case of c-PTIO, the number of live late larvae/larvae during tail regression and juveniles were counted after 24 h of treatment and expressed as 100% of total individuals, as previously reported [9], [14]. Treated larvae were allowed to develop to the desired stage and then directly observed or collected by low speed centrifugation after washing in PBS buffer. The pellets were stored at −80°C until use and subsequently used for protein extraction, NO detection and RNA preparation.

### Protein extraction

Larval pellets (50–100 mg) were homogenized in two volumes of RIPA lysis buffer (150 mM NaCl, 50 mM Tris-HCl pH 7.6, 5 mM EDTA, 0.5% NP-40, 0.5% sodium deoxycholate, 0.1% SDS) supplemented with protease inhibitors (1 mM PMSF and Complete Protease Inhibitor Cocktail Tablets, Roche) and phosphatase inhibitors (PhosSTOP Cocktail Tablets, Roche). After centrifugation, protein concentration was determined using a Bio-Rad Protein Assay Reagent (Bio-Rad) with bovine serum albumin as a standard.

### Fluorimetric determination of NO concentration

Larval pellets (50–60 mg) were homogenized in PBS and sonicated. The samples were centrifuged at 13,500 g for 5 min at 4°C and the supernatants were collected for NO analysis using 2,3-diaminonaphthalene (DAN) to form the fluorescent product 1-(*H*)-naphthotriazole. Briefly, samples (80 µl) were incubated at 26°C with nitrate reductase 0.06 U/ml, FAD 2.5 µM and NADPH 100 µM final for 1 hour. Then, 10 µl of DAN (0.05 mg/ml in 0.62 M HCl) was added and the samples were incubated for 20 min in the dark. After stabilization with 1N sodium hydroxide, formation of 1-(*H*)-naphthotriazole was measured using a spectrofluorimeter (Shimadzu RF-5301 PC) with excitation and emission at 365 and 425 nm, respectively. Calibration curves were performed daily with sodium nitrite (1–10 µM) in PBS. The experiments were carried out on three biological replicates.

### Western blot analysis

Larva lysates were prepared as previously described [14] and examined by 10% SDS-PAGE. The gel transferred to nitrocellulose membrane was analyzed with antibodies against p44/42 MAP Kinase (ERK 1/2) (ERK) (1∶1000) (Cell Signaling), Phospho-p44/42 MAPK (ERK1/2) (P-ERK) (1∶500) (Cell Signaling). After washing in PBS with 0.1% tween, labeled proteins were detected by ECL PLUS (ERK) and Supersignal West Pico Chemiluminescent Substrate (Pierce) (P-ERK). Densitometric analyses were carried out on scanned western blot images, using ImageJ programme.

### Bioinformatic analysis

*Ciona* transcripts were retrieved from Aniseed (http://www.aniseed.cnrs.fr/). The identification of mkp1 and mkp3 was performed analyzing all the transcripts coding for dual specific phosphatases existing in *Ciona* transcriptome by blastp programme to find homology from other sources. Multiple sequence alignments of *C. intestinalis* mkp1 and mkp3 proteins with homologous counterparts were carried out by Clustal W.

### RNA extraction and cDNA synthesis

Total RNA was extracted at different developmental stages using TRIzol (Invitrogen) according to the manufacturer's instructions. Briefly, extraction with chloroform/isoamyl alcohol (24∶1) was performed following RNA precipitation by addition of glycogen and isopropyl alcohol. Contaminating DNA was degraded by treating each sample with DNase (Roche) and finally removing the enzyme with RNeasy MinElute Cleanup Kit (Qiagen). The amount of total RNA was estimated by the absorbance at 260 nm and the purity by 260/280 and 260/230 nm ratios, by Nanodrop (ND-1000 UV-Vis Spectrophotometer; NanoDrop Technologies). The integrity of RNA was checked in agarose gel electrophoresis by visualizing intact rRNA subunits (28S and 18S). For each sample, 1 µg of total RNA was retrotranscribed with iScript cDNA Synthesis kit (Bio-Rad), following the manufacter's instructions. cDNA was diluted 1∶10 with H_2_O prior to use in Real Time qPCR experiments.

### Real Time qPCR

Data from each cDNA sample were normalized using ribosomal protein R27a as reference gene, whose levels remained relatively constant in all the developmental stages examined. The gene sequences were retrieved from Aniseed (http://www.aniseed.cnrs.fr/). For each gene, specific primers were designed on the basis of nucleotide sequence with the help of Primer 3 (see Table 1), except for *dhg* and *mkp1* for which we used the primers reported in literature [11]. The amplified fragments using Taq High Fidelity PCR System (Roche) were purified from agarose gel using QIAquick Gel extraction kit (Qiagen) and specificity of PCR products was checked by DNA sequencing (Molecular Biology Service, SZN, Naples). Specificity of amplification reactions was verified by melting curve analysis. The efficiency of each primer pair was calculated according to standard methods curves using the equation E = 10^−1/slope^. Five serial dilutions were set up to determine Ct values and reaction efficiencies for all primer pairs. Standard curves were generated for each oligonucleotide pair using the Ct values versus the logarithm of each dilution factor. PCR efficiencies were calculated for control and target genes and were found to be 2 in most of them, only for *vwa1c* was 1.985 and for *mkp*3 1.926. Diluted cDNA was used as a template in a reaction containing a final concentration of 0.3 µM for each primer and 1 X FastStart SYBR Green master mix (total volume of 10 ul). PCR amplifications were performed in a ViiATM 7 Real Time PCR System (Applied Biosystems) thermal cycler using the following thermal profile: 95°C for 10 min, one cycle for cDNA denaturation; 95°C for 15 sec and 60°C for 1 min, 40 cycles for amplification; 72°C for 5 min, one cycle for final elongation; one cycle for melting curve analysis (from 60°C to 95°C) to verify the presence of a single product. Each assay included a no-template control for each primer pair. To capture intra-assay variability all Real Time qPCR reactions were carried out in triplicate. Fluorescence was measured using ViiATM 7 Software (Applied Biosystems). The expression of each gene was analyzed and internally normalized against R27a using REST software (Relative Expression Software Tool) based on Pfaffl method [17], [18]. Relative expression ratios equal or greater than ±2 were considered significant. Experiments were repeated at least three times.

**Table 1:** Features of transcripts used for gene expression analysis: accession numbers, primer sequences, length of PCR amplified fragments and data on gene regulation and localization in larvae.

Gene Acronym	Accession Number	Gene Name	Gene Regulation	Localization	Primer sequences (5′-3′)	Fragment bp
*R27a*	KYOTOGRAIL2005.209.6.1	Ribosomal protein	reference	Ubiquitous	For-ATCCACCCTTCACCTTGTG	156
					Rev-GGAGATCTTGCCATTTTCA	
*dhg*	ci0100130315	Dehydrogenase	down-regulated by ERK [11]	Ubiquitous	For-GTCACCGTTTCCTCTGAAGC	299
					Rev-GCGCCGTGTATTATGGTCTT	
*ets*	ci0100152617	Ets-related transcription factor	up-regulated by ERK [11]	Papillae [11]	For-GACGAAGCATGTGTACCTGAC	228
					Rev-GCGGTTTTTCTGCACACCCC	
*mkp1*	ci0100138796	Dual specific MAPK phosphatase 1	down-regulated by NO *		For-CCACTTTCCAGACCGATTTC	401
					Rev-CCTCACAGGTCCACTCCATT	
*mkp3*	ci0100140262	Dual specific MAPK phosphatase 3	down-regulated by NO *		For-CGATGTTGGCGTTGCTGTAC	164
					Rev-GATGGACGGAGCATGAATGG	
*mx*	ci0100149359	Matrix metalloprotease	up-regulated by ERK [11]	Papillae [11]	For-GATTCCCAGCTAGTATCCG	263
					Rev-CGTTCTTCTGCTTGGATTGT	
*NOS*	ci0100153759	Nitric oxide synthase		Palps and tail [9]	For-AGAGTGAAAGCCTGTCGCATA	150
					Rev-AACCAATGCGGTGGTTGTAG	
*rev-erb*	ci0100143297	Steroid hormone receptor	down-regulated by NO *	Palps [33]	For-CAGATCCCATTGCCTGAGTTG	152
					Rev-CAGTCTATTTGGTCGTCGTCG	
*srf*	ci0100151810	Serum responsive factor			For-CGTACTGTTGTTATGGCTG	239
					Rev-GTAGCTAACTCATGCGCC	
*vwa1c*	ci0100142643	Von Willebrand factor	down-regulated by ERK [11]	Papillae [11]	For-GTATGTCAACATGTAGCATCG	214
					Rev-CACACGCTTCCTATAGACCTC	

*This work.

### Statistical analysis

Statistical analysis was performed using GraphPad Prism version 4.00 for Windows (GraphPad Software, San Diego California USA). The results of morphological and biochemical experiments were reported as means ± SEM and analyzed by unpaired *t*-test for comparison between the groups. P<0.05 was considered statistically significant. For Real Time qPCR analysis, results were reported as means ± SD and significance was tested using the “Pair Wise Fixed Reallocation Randomisation Test”, developed by REST software [17], [18]. The number of experiments was reported in the figure legends.

## Results

### NO affects ERK phosphorylation

To characterize the involvement of NO during metamorphosis, hatched larvae were treated with different compounds to modulate endogenous NO levels. In details, to reduce endogenous NO levels, two approaches were used. Larvae were treated with TRIM, the specific NOS inhibitor which interferes with the binding of both L-arginine and the cofactor BH_4_, and with the scavenger c-PTIO, which directly reacts with NO [16]. To increase NO levels, larvae were treated with the slow-releasing NO donor sperNO. To confirm the effectiveness of these treatments, the endogenous NO levels were measured by monitoring nitrite formation. After 6 h of TRIM (250 µM) treatment, corresponding to 24 hpf, the basal concentration of NO significantly decreased from 3.14±0.30 to 1.14±0.13 nmol nitrite/mg protein (*P* = 0.0005). The NO scavenger c-PTIO (300 µM) exhibited the same effect as TRIM, resulting in a decrease of NO levels from 2.36±0.29 to 1.2±0.19 nmol nitrite/mg protein (*P* = 0.0043), at 22 hpf, after 4 h of treatment. In the presence of sperNO (250 µM) after 6 h, corresponding to 24 hpf, the level of NO increased from 4.26±0.44 to 6.15±0.31 nmol nitrite/mg protein (*P* = 0.0037).

**Figure 1:**
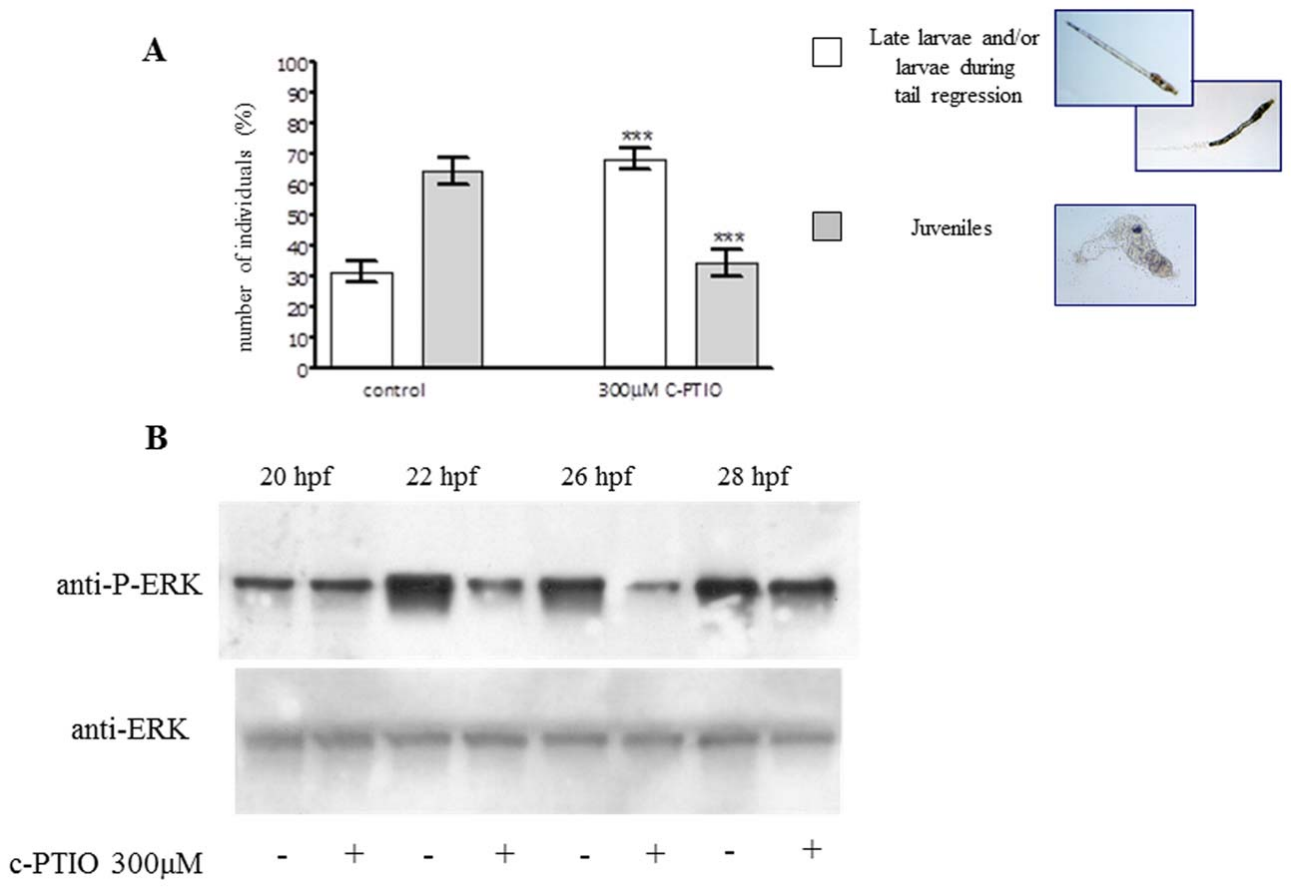
Decrease of endogenous NO levels with the NO scavenger c-PTIO results in a slow-down of metamorphosis and a reduction of ERK phosphorylation. (A) Hatched larvae were treated with c-PTIO (300 µM) and after 24 h the number of late larvae, larvae during tail regression and juveniles were counted and reported as percent of the total. Results are representative of 10 independent experiments. Data, expressed as means ± SEM, are assessed by unpaired *t*-test. Asterisk represents the significance respect to the control ***P<0.001 (white bar <0.0001, grey bar  = 0.0002). (B) Hatched larvae treated with c-PTIO were examined at different times for ERK activation. Representative experiment showing the western blot analyzed with anti-P-ERK and anti-ERK antibodies.

The modulation of endogenous NO levels affected the rate of metamorphosis. Indeed, hatched larvae treated with c-PTIO showed a slow-down of metamorphosis, as evidenced by the increase of the percentage of late larvae or larvae during tail regression to 69% with respect to 32% in controls and a concomitant decrease of juveniles to 35% with respect to 65% in controls, observed after 24 h treatment (Figure 1A). Higher levels of c-PTIO proved to be toxic. Also TRIM and sperNO have been recently shown to affect the rate of metamorphosis. In particular, the NOS inhibitor caused a slow-down and the NO donor an acceleration of the process [14].

**Figure 2:**
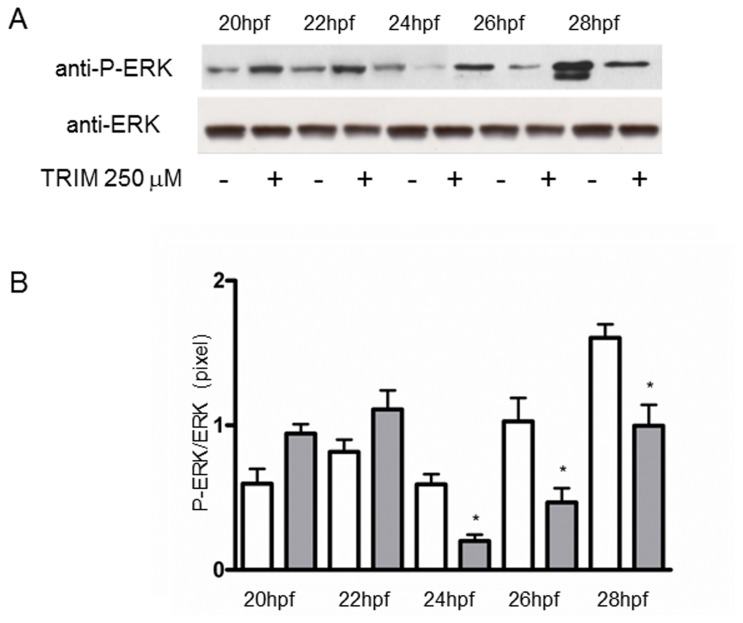
Decrease of ERK phosphorylation by NOS inhibitor TRIM. Hatched larvae were treated with TRIM (250 µM) and samples at different times were examined for ERK activation. (A) Representative experiment showing the western blot analyzed with anti-P-ERK and anti-ERK antibodies. (B) Histogram showing densitometric analysis of immunopositive P-ERK respect to ERK bands. In control sample at 28 hpf the analysis is performed on the upper band. White bars, without TRIM; grey bars, with TRIM. Results are representative of 5 independent experiments. Data, expressed as means ± SEM, are assessed by unpaired t-test. Asterisk represents the significance respect to the control *P<0.05 (24 hpf  = 0.01; 26 hpf  = 0.042; 28 hpf  = 0.024).

**Figure 3:**
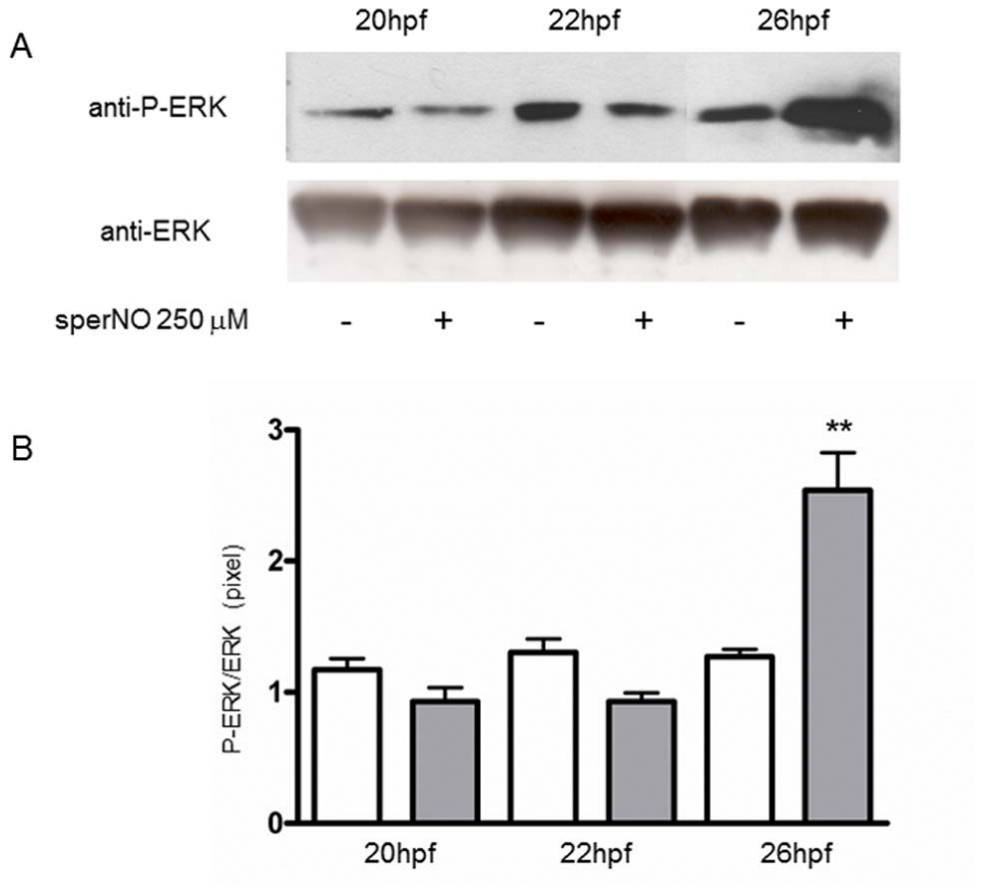
Increase of ERK phosphorylation by NO donor sperNO. Hatched larvae were treated with sperNO (250 µM) and samples at different times were examined for ERK activation. (A) Representative experiment showing the western blot analyzed with anti-P-ERK and anti-ERK antibodies. (B) Histogram showing densitometric analysis of immunopositive P-ERK respect to ERK bands. White bars, without sperNO; grey bars, with sperNO. Results are representative of 3 independent experiments. Data, expressed as means ± SEM, are assessed by unpaired *t*-test. Asterisk represents the significance respect to the control **P<0.01 (0.005).

Larvae treated with the different compounds c-PTIO, TRIM and sperNO were withdrawn at different times after treatment and examined for ERK activation. The NO scavenger c-PTIO induced the decrease of ERK phosphorylation at 22 and 26 hpf, corresponding to 4 and 8 h of treatment, whereas a substantial amount of P-ERK was produced at 28 hpf (Fig. 1B). Also TRIM reduced ERK phosphorylation (Figure 2A). The densitometric analysis of the P-ERK immunopositive bands respect to the ERK bands revealed a significant reduction of ERK phosphorylation at 24, 26 and 28 hpf, whereas at shorter times of 20 and 22 hpf, corresponding to 2 h and 4 h of treatment, the differences between control and treated larvae were not statistically significant (Figure 2B). Treatment of hatched larvae with the slow NO donor sperNO resulted in a significant increase of P-ERK at 26 hpf, corresponding to 8 h of treatment (Figure 3A), as confirmed by densitometric analysis of the P-ERK immunopositive bands respect to constitutive ERK (Figure 3B).

### NO regulated gene expression

**Figure 4:**
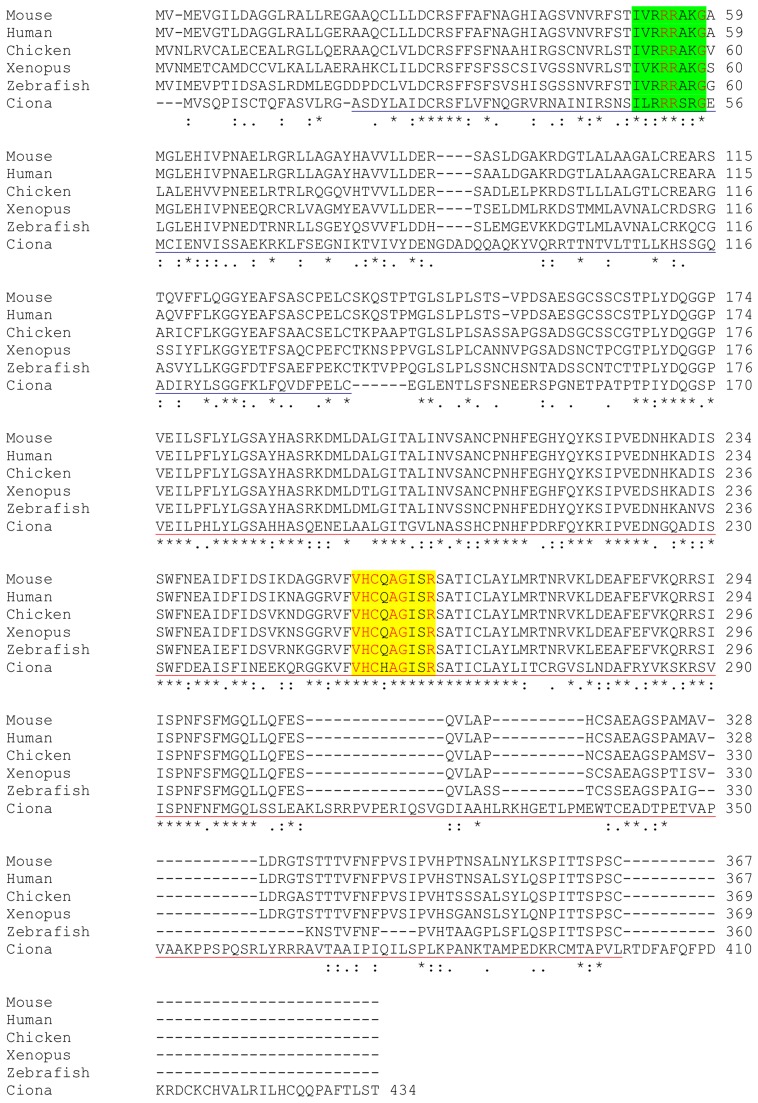
Multiple sequence alignment of *C. intestinalis* mkp1 with homologous counterparts. *Ciona* sequence ci0100138796 was aligned using ClustalW with the following sequences retrieved from Uniprot database: zebrafish (*Danio rerio*) Q6IQU5, *Xenopus laevis* Q91790, chicken (*Gallus gallus*) F1NPN1, human P28562 and mouse P28563. The MAPK binding domain (MKB) or N-terminal domain is underlined in blue (21–136 aa in human sequence), the dual specific phosphatase domain (DSP) or C-terminal domain is underlined in red (175–367 aa in human sequence). The highly conserved catalytic motif I/VHCXAGXXR in DSP domain is highlighted in yellow, and the motif -ΨΨXRRΨXG- in the MKB domain is highlighted in green. Ψ is a hydrophobic residue and X is any residue. In red are the highly conserved positions including the catalytic cysteine (C 258 in human).

To investigate if NO affected also gene transcription related to ERK activation, we selected a series of ERK target genes mostly expressed in territories specialized for metamorphosis, such as palps and/or papillae, where NOS expression, NO production and ERK activation occur [9], [11]. These genes include: dehydrogenase (*dhg*), ets-related transcription factor (*ets*), matrix metalloprotease-24-precursor (*mx*), nuclear hormone receptor (*rev-erb*), and the von Willebrand factor (*vwa1c*). We also selected serum response factor (*srf*) and *NOS*, whose expression in other systems has been shown to be regulated by ERK activation [19], [20] (table 1). Finally, we considered dual specific MAP kinase phosphatases (mkps) which regulate the cellular levels of activated ERK through dephosphorylation of specific threonine and tyrosine residues [21]. By *in silico* analysis of the *Ciona* transcriptome (http://www.aniseed.cnrs.fr/), we identified two transcripts (ci0100138796, ci0100140262) belonging to type II of the dual specific phosphatase family, containing both the dual specific phosphatase domain and the N-terminal MAPK binding domain. Multiple sequence alignments of these proteins with homologous counterparts highlighted that they exhibit a significant homology with mkp1 (Figure 4) and mkp3 enzymes (Figure S1), respectively.

**Figure 5:**
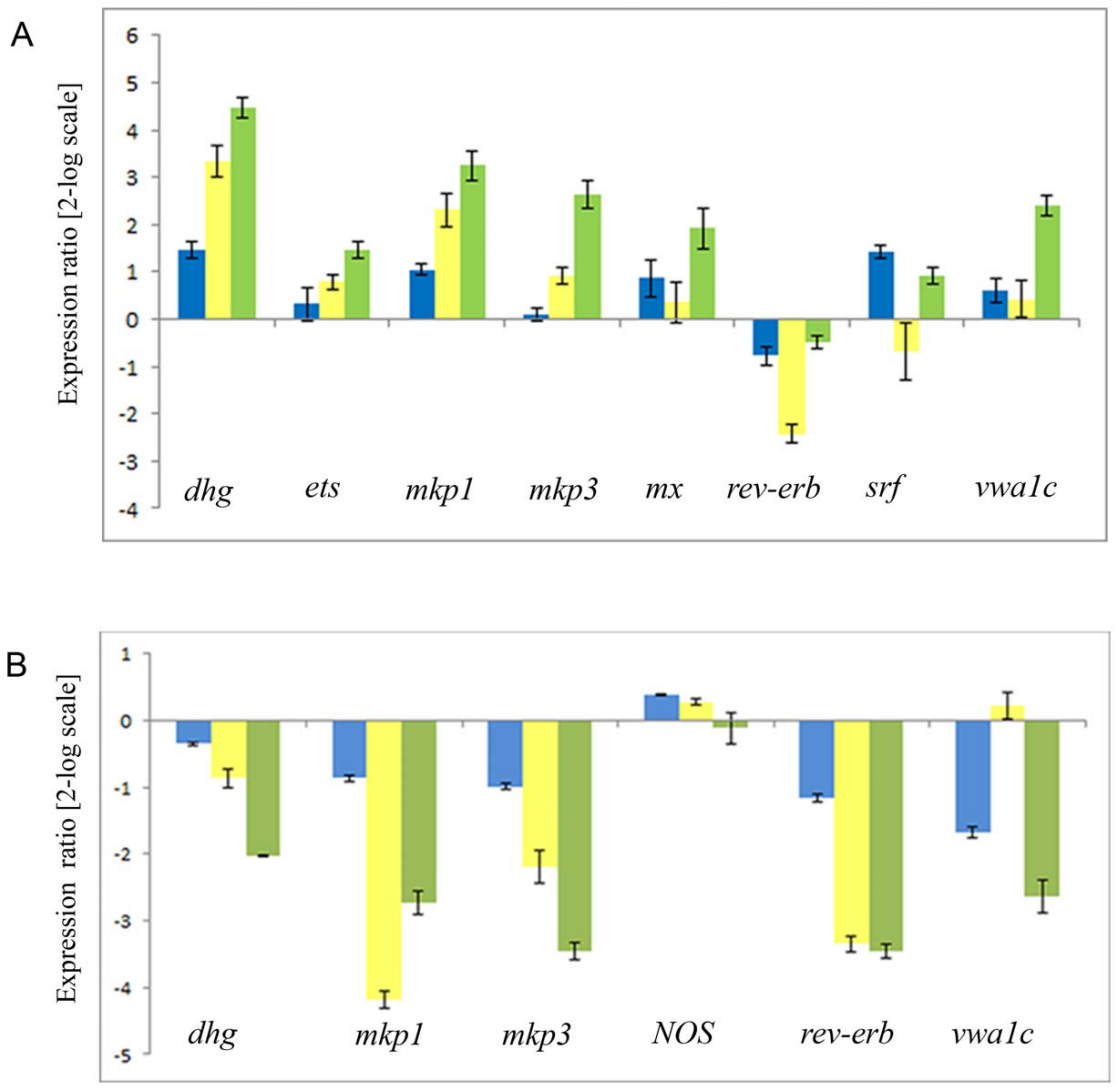
Gene regulation in response to NO levels during larval development. Histogram shows the differences in expression levels of analyzed genes, followed by Real Time qPCR. Hatched larvae (18 hpf) incubated with 250 µM TRIM (A) or 250 µM sperNO (B) were collected at 22 hpf (blue), 24 hpf (yellow) and 26 hpf (green). Data are reported as a fold difference compared to the control, larvae in sea water without TRIM or sperNO (means ± SD). Values equal or greater than ±2 were considered significant. The experiments were repeated at least 3 times.

The relative expression of all the above mentioned genes was followed by Real Time qPCR experiments, after treating hatching larvae (18 hpf) with the NOS inhibitor TRIM to reduce endogenous NO levels (Figure 5A). Larvae were collected at 22, 24 and 26 hpf corresponding to 4, 6 and 8 h of treatment. The expression of *dhg* and *vwa1c* increased after 6–8 h and 8 h of treatment, respectively, whereas *rev-erb* was down-regulated at 6 h. The expression of *mkp1* and *mkp3* increased with respect to the control sample. In detail, the increase of expression of *mkp1* already started at 24 hpf after 6 h of treatment, while the beginning of the increase of *mkp3* expression occurred at 26 hpf after 8 h of treatment. The expression of the other genes, *ets*, *mx* and *srf* was unaffected. To confirm that NO is responsible for the changes in gene expression of *dhg*, *mkp1*, *mkp3*, *rev-erb* and *vwa1c*, hatched larvae were treated with the slow NO donor sperNO. Under these conditions, also the expression of *NOS* was investigated to verify a possible feedback by NO, as recently reported in another ascidian [22]. Samples, collected at 22, 24, 26 hpf after 4, 6 and 8 h of treatment, were analyzed for quantitative gene expression (Figure 5B). *Mkp1*, *mkp3*, *dhg*, *rev-erb* and *vwa1c* were down-regulated in the presence of sperNO, with respect to the control. In detail, the down-regulation of *mkp1* and *rev-erb* reached 4 fold and 3 fold, respectively, at 24 hpf after 6 h of treatment. *Dhg*, *mkp3* and *vwa1c* were down-regulated between 2 fold and more than 3 fold, at 26 hpf after 8 h of treatment. The expression of *NOS* was not affected by increasing NO levels.

**Figure 6:**
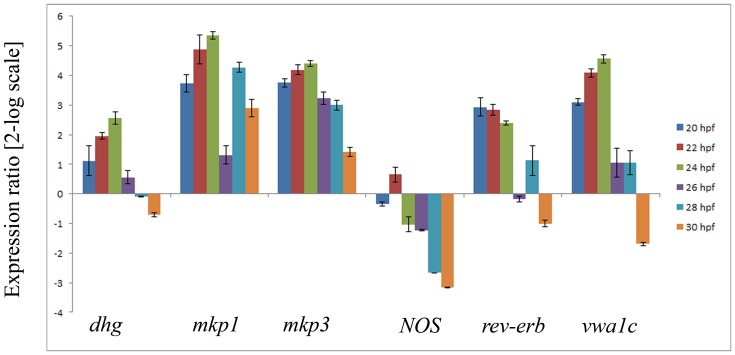
Gene expression profiles during larval development. Histogram shows the values of gene expression at different times of larval development respect to those obtained for hatching larvae (18 hpf), followed by Real Time qPCR. Data are reported as a fold difference compared to the control (means ± SD). Values equal or greater than ±2 were considered significant. The experiments were repeated at least 3 times.

The expression of the NO-regulated genes was followed during larval development at different times from hatching (18 hpf) to the onset of metamorphosis (28–30 hpf) (Figure 6). The expression of ERK target genes *dhg* and *vwa1c* was maximum at 22–24 hpf, whereas *rev-erb* was up-regulated between 20 and 24 hpf. The expression of *NOS* was approximately constant until 26 hpf, while it was down-regulated at 28 and 30 hpf. The gene expression pattern of *mkp1* exhibited two peaks, the first one at 22–24 hpf and the second one at 28–30 hpf. On the contrary, the expression profile of *mkp3* remained high from 20 to 28 hpf.

### NO regulates ERK pathway through mkps expression

**Figure 7:**
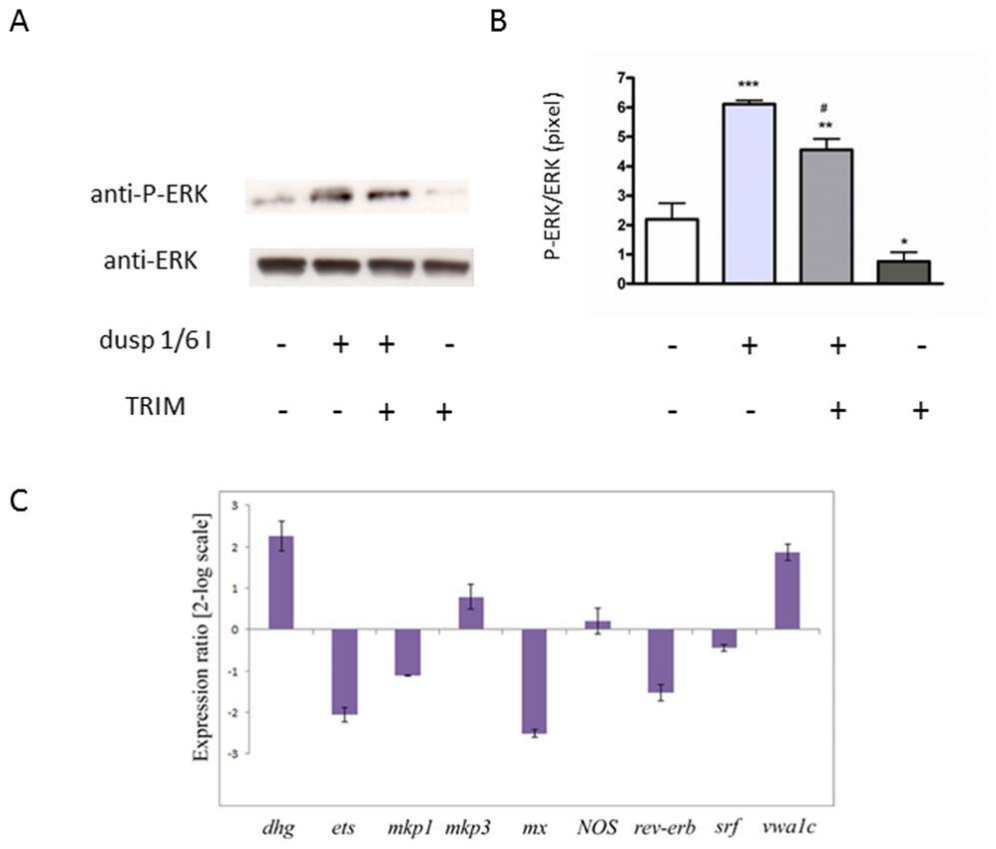
ERK independent mkp expression by NO regulates ERK activation. (A) Hatched larvae were treated with 0.25 µM of dusp 1/6 I (+) in the absence or presence of TRIM (250 µM). Samples collected after 6 h of treatment were examined for ERK activation. Representative western blot analyzed with anti-P-ERK and anti-ERK antibodies. (B) Data of densitometric analyses of 3 independent experiments are reported as means ± SEM and assessed by unpaired *t*-test. Asterisk represents the significance respect to the control *P<0.05 (0.049), **P<0.01 (0.007), ***P<0.001 (0.0002). # represents the significance respect to the TRIM alone (*P* = 0.0008). (C) Gene regulation in response to MEK inhibitor U0126. Histogram shows the values of gene expression at 6 h treatment respect to the untreated larvae, followed by Real Time qPCR. Data are reported as a fold difference compared to the control (means ± SD). Values equal or greater than ±2 were considered significant. The experiments were repeated at least 3 times.

To confirm that mkp1 and mkp3 specifically dephosphorylate ERK during *C. intestinalis* larval development, we treated larvae at hatching with the dual specificity protein phosphatase 1/6 inhibitor (dusp 1/6 I), a cell-permeable compound that inhibits phosphatase activity of mkp1 and mkp3 in HeLa cells. As you can see from Figure 7A,B, ERK phosphorylation increased in the presence of the mkps inhibitor. Moreover, larvae at hatching were first treated with the NOS inhibitor TRIM, which up-regulates the expression of *mkps* and causes the reduction of P-ERK, and then with dusp 1/6 I, to inhibit the activity of the new produced mkps. Under these conditions, the levels of P-ERK are significantly increased with respect to the control and TRIM alone (Figure 7A,B). This suggested that the NO-mediated up-regulation of *mkps* led to the production of active phosphatases, thus affecting ERK phosphorylation. Finally, to understand if P-ERK, on its turn, can modulate *mkps* transcription, their expression was followed after ERK inhibition by the MEK inhibitor U0126. Larvae at hatching were treated with the inhibitor resulting in a complete block of metamorphosis after 24 h treatment, in agreement with Chambon et al., 2007 [11]. The expression of *mkp1* and *mkp3* was not significantly affected by the treatment (Figure 7C). The same result was obtained for *NOS*, *srf* and *rev-erb*. On the other hand, the expression of the previously identified ERK target genes was regulated as expected [11]. Indeed, *dhg* and *vwa1c* were up-regulated and *ets* and *mx* were down-regulated, as reported by Chambon et al., 2007 [11].

## Discussion

Metamorphosis of marine organisms represents an ancient evolutionary advantageous process because it matches the reproductive state of the adult with the presence of favorable environmental factors, such as nutrients availability. Generally, a reduction in amino acids availability can affect NO signaling, because L-arginine, the most nitrogen-rich amino-acid is the main substrate of NOS. Indeed, it has been previously reported that nitrogen deprivation triggers alternate reproductive states in many unicellular organisms [2] and that NO can negatively or positively affect metamorphosis in a variety of taxa, including ascidians [9], [22]–[27].

The results of the present study add new insights into the molecular mechanisms affected by NO during *Ciona* metamorphosis. Interestingly, we have demonstrated that changes in endogenous NO levels in larvae affects a key event necessary for tail retraction and subsequent metamorphosis, i.e. ERK activation. Indeed, when NO levels decrease, a reduction of ERK phosphorylation occurs. On the contrary, when the NO levels rise, ERK activation increases. As expected, a decrease or increase of ERK phosphorylation affects the rate of metamorphosis, with a consequent delay or acceleration of the process, respectively. The finding that after 10 h treatment the decrease of ERK phosphorylation by TRIM persisted, whereas with c-PTIO the level of ERK phosphorylation became comparable to that of the control, might reflect the different mechanism of action of the two compounds. It is reasonable to hypothesize that TRIM, which specifically inhibits NO production, continuously reduces NO levels, whereas the NO scavenger is depleted during the experimental time and cannot react anymore with NO newly produced by NOS [16].

During *Ciona* larval development, the first peak of ERK activation in the papillae (20–22 hpf) is responsible to activate a series of downstream genes, likely involved in the acquiring of competence and the attachment of larvae to the substrate. Indeed, we have demonstrated that changes in NO levels also affect the expression of ERK-target genes, including the metabolic enzyme dehydrogenase, *dhg*, and the developmental genes, *vwa1c* and *rev-erb*, whereas other genes such as *ets*, *mx*, *srf* and *NOS* are unaffected. Vwa domains are present in extracellular matrix molecules, adhesion proteins and cell surface receptors [28] and are also found in complement factors [29]. In the ascidian *Boltenia villosa*, *vwa1* is expressed during metamorphosis at competence acquisition [30]. In *Ciona*, *vwa1c* is down-regulated by ERK activation and is expressed in papillae [11]. Our finding that the maximum expression of *vwa1c* is at 20–24 hpf, when the formation of palps and papillae occurs and the larva acquires the ability to sense external stimuli, suggests the involvement of this factor in the structural reorganization of papillae and possibly in the maturation of adult immune system and in re-structuring of larval tissues during metamorphosis [30], [31]. Regarding rev-erb, this is a nuclear receptor involved in circadian rhythm in vertebrates. In *Ciona*, circadian rhythms have been characterized at physiological and molecular levels, although no data are available on the processes regulated by circadian clock [32]. In *Ciona,* rev-erb is localized in the palps [33]. Our finding that the maximum expression of this receptor occurs at 20–24 hpf is relevant considering that at this time larvae become light sensitive [34], [35]. The regulation of *rev-erb* by NO has also been reported in other systems [36]. However, the down-regulation of *rev-erb* both in the presence of NOS inhibitor and NO donor suggests that, besides ERK activation, NO can mediate other ERK-independent pathways leading to *rev-erb* expression regulation. The fact that the previously characterized ERK target genes, *ets* and *mx*, are not affected by our pharmacological approach could be ascribed to the lower abundances of their transcripts, which fail to give detectable differences.

Regarding the molecular mechanisms that modulate ERK activation, a key role is played by the interaction between kinases and phosphatases [37]. Different types of mkps have been reported, whose substrate specificity towards MAP kinases is dependent on the system and cell type [21], [38], [39]. By *in silico* analysis of *Ciona* genome, two transcripts, encoding for proteins containing all the characteristic domains responsible for ERK interaction and dephosphorylation, have been characterized. These two transcripts show significant similarity with mkp1 and mkp3 from different sources. Our results on mkps inhibition confirm that mkp1 and mkp3 are indeed specific phosphatases of ERK during *Ciona* development. Moreover, the correlation between the temporal expression of *mkp1* and the profile of ERK activation suggests that mkp1 is the phosphatase responsible for ERK-dependent gene transcription. Indeed, mkp1 family members are usually localized in the nucleus [40], where ERK translocates for downstream gene transcription. On the contrary, mkp3 is usually localized in the cytoplasm [40], where it could dephosphorylate ERK, thus affecting its cytoplasmic substrates.

An interesting outcome of this paper is that NO regulates mkps expression affecting finally ERK pathway. Indeed, the reduction of endogenous NO levels contributes to the up-regulation of *mkp1* and *mkp3* expression and to the production of active mkps with consequent ERK dephosphorylation. On the other hand, the increase of NO levels causes the down-regulation of these transcripts with consequent ERK activation. The decrease of *mkp*s expression by NO has also been observed in endothelial cells and it has been ascribed to destabilization of *mkp-3* mRNA [41]. Our finding that ERK inhibition by MEK inhibitor does not affect *mkps* mRNA levels suggests that regulation of *mkps* expression by NO occurs through an ERK-independent pathway. Indeed, in different cell types, mkps induction can be either dependent or independent on ERK activation [42]–[44].

The results reported in this paper contribute to expand existing data on the role of NO on metamorphosis in a variety of taxa, including chordates, echinoderms, mollusks and annelids. In the ascidians *Boltenia villosa* and *Cnemidocarpa finmarkiensis*, the echinoderm *Lytechinus pictus*, the gastropod mollusks *Ilyanassa obsoleta* and *Crepidula fornicata* and the polychaete annelid *Capitella teleta*, NO has been reported to be a negative regulator of metamorphosis [23]–[27]. On the contrary, in the ascidian *Herdmania momus* NO has been recently shown to act as a positive regulator, inducing metamorphosis [22]. However, the picture emerging from literature is complex and in some cases the effect on metamorphosis seems to be dependent on the compound used, likely due to the different modulation of endogenous NO levels [9], [14], [45]. Therefore, a fine regulation of NO levels could differently affect the same biological process. In this study, we have effectively measured the NO levels following treatments with a NOS inhibitor, a NO scavenger and a NO donor and we have demonstrated that NO positively influences the rate of larval development by affecting ERK signaling. The constant expression of *NOS* during larval development until 26 hpf suggests that the modulation of endogenous NO levels could rely at these stages on the regulation of the enzyme activity by intracellular calcium concentration [46]. On the contrary, the marked decrease of *NOS* expression at 28–30 hpf, when larvae are attached to the substrate and metamorphosis starts, suggests that at this stage NO production could be regulated at the transcriptional level. The reduction of *NOS* expression when metamorphosis progresses, has also been reported in other organisms [22], [47]–[48]. Overall our findings suggest that during larval development NO induces ERK signaling, necessary for acquiring of competence, larval settlement and the initiation of the apoptotic wave. Immediately after, NO is likely no more necessary and therefore *NOS* expression decreases.

## Conclusions

This study provides new insights into the role played by NO in regulating key molecular mechanisms responsible for life cycle transitions. We demonstrate that during *Ciona* larval development, changes in endogenous NO levels lead to an ERK-independent mkps regulation. These phosphatases contribute to maintain ERK phosphorylation levels and consequent transcription of downstream genes involved in key metamorphic events, such as competence acquisition and re-construction of larval tissues.

## Supporting Information

Figure S1 **Multiple sequence alignment of** ***C. intestinalis*** **mkp3 with homologous counterparts.** *Ciona* sequence ci0100140262 was aligned using ClustalW with the following sequences retrieved from Uniprot database: zebrafish (*Danio rerio*) Q7T2L8, *Drosophila melanogaster* Q9VVW5, chicken (*Gallus gallus*) Q7T2L9, Mouse Q9DBB1. The MKB or N-terminal domain is underlined in blue (22–149 aa in *D. melanogaster*), the DSP or C-terminal domain is underlined in red (215–357 aa in *D. melanogaster*). The catalytic motif I/VHCXAGXXR in DSP domain is highlighted in yellow, and the motif -ΨΨXRRΨXXG- in the MKB domain is highlighted in green. Ψ is a hydrophobic residue and X is any residue. In red are the highly conserved positions including the catalytic cysteine (C 302 in *D. melanogaster*). Finally the poly-his 201–209 present in *D. melanogaster* but not conserved in *ciona* and homologous counterparts is highlighted in magenta.
(TIF)
