# Supplementary figures and images for: Nitric Oxide Affects ERK Signaling through Down-Regulation of MAP Kinase Phosphatase Levels during Larval Development of the Ascidian Ciona intestinalis
Source: PLoS One. 2014 Jul 24;9(7):e102907. doi: 10.1371/journal.pone.0102907 (PMC4109947; doi:10.1371/journal.pone.0102907)

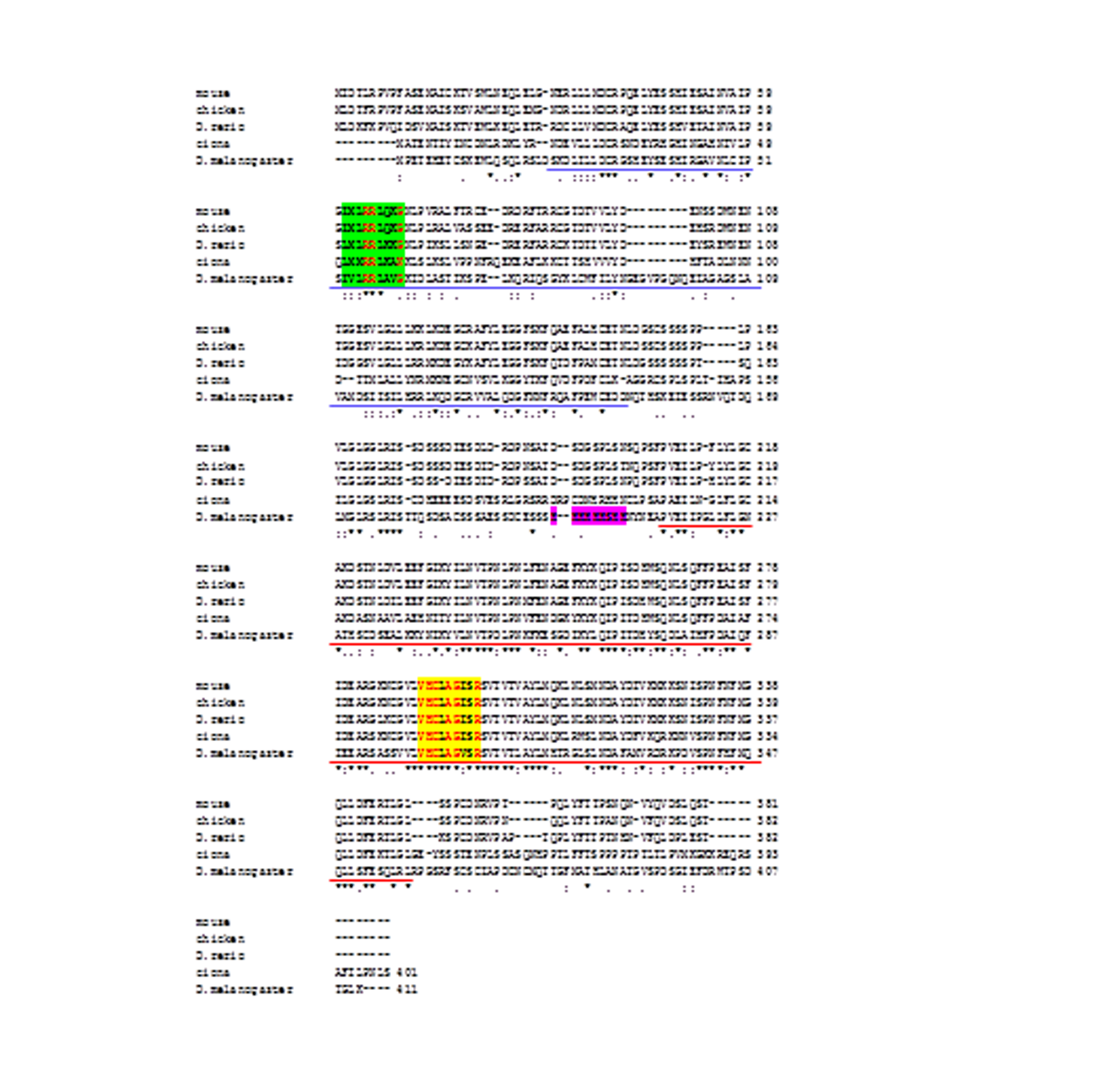

Supplement: Figure S1 — Multiple sequence alignment of C. intestinalis mkp3 with homologous counterparts. Ciona sequence ci0100140262 was aligned using ClustalW with the following sequences retrieved from Uniprot database: zebrafish (Danio rerio) Q7T2L8, Drosophila melanogaster Q9VVW5, chicken (Gallus gallus) Q7T2L9, Mouse Q9DBB1. The MKB or N-terminal domain is underlined in blue (22–149 aa in D. melanogaster), the DSP or C-terminal domain is underlined in red (215–357 aa in D. melanogaster). The catalytic motif I/VHCXAGXXR in DSP domain is highlighted in yellow, and the motif -ΨΨXRRΨXXG- in the MKB domain is highlighted in green. Ψ is a hydrophobic residue and X is any residue. In red are the highly conserved positions including the catalytic cysteine (C 302 in D. melanogaster). Finally the poly-his 201–209 present in D. melanogaster but not conserved in ciona and homologous counterparts is highlighted in magenta. (TIF) [file pone.0102907.s001.tif]
